# Effect of Automated Closed-loop ventilation versus convenTional VEntilation on duration and quality of ventilation in critically ill patients (ACTiVE) – study protocol of a randomized clinical trial

**DOI:** 10.1186/s13063-022-06286-w

**Published:** 2022-04-23

**Authors:** Michela Botta, Anissa M. Tsonas, Jante S. Sinnige, Ashley J. R. De Bie, Alexander J. G. H. Bindels, Lorenzo Ball, Denise Battaglini, Iole Brunetti, Laura A. Buiteman–Kruizinga, Pim L. J. van der Heiden, Evert de Jonge, Francesco Mojoli, Chiara Robba, Abraham Schoe, Frederique Paulus, Paolo Pelosi, Ary Serpa Neto, Janneke Horn, Marcus J. Schultz, The ACTiVE collaborative group, Michela Botta, Anissa M. Tsonas, Jante S. Sinnige, Ashley J. R. De Bie, Alexander J. G. H. Bindels, Lorenzo Ball, Denise Battaglini, Iole Brunetti, Laura A. Buiteman–Kruizinga, Pim L. J. van der Heiden, Evert de Jonge, Francesco Mojoli, Chiara Robba, Abraham Schoe, Frederique Paulus, Paolo Pelosi, Ary Serpa Neto, Janneke Horn, Marcus J. Schultz

**Affiliations:** 1 Department of Intensive Care, Amsterdam UMC location University of Amsterdam, Meibergdreef 9, Amsterdam, The Netherlands; 2 Department of Intensive Care, Catharina Hospital Eindhoven, Eindhoven, The Netherlands; 3 Department of Anesthesia and Intensive Care, San Martino Polyclinic Hospital, IRCCS for Oncology and Neurosciences, Genova, Italy; 4 Department of Intensive Care, Reinier de Graaf Hospital, Delft, The Netherlands; 5 Department of Intensive Care, Leiden University Medical Centre, Leiden, The Netherlands; 6 Department of Anesthesia and Intensive Care, San Matteo Polyclinic Foundation, University of Pavia, Pavia, Italy; 7 Faculty of Health, ACHIEVE, Centre of Applied Research, Amsterdam University of Applied Sciences, Amsterdam, The Netherlands; 8 Department of Surgical Sciences and Integrated Diagnostic (DISC), University of Genova, Genova, Italy; 9 Australian and New Zealand Intensive Care Research Centre, School of Public Health and Preventive Medicine, Monash University, Melbourne, Australia; 10 Department of Critical Care Medicine, Hospital Israelita Albert Einstein, São Paulo, Brazil; 11 Amsterdam Neuroscience, Amsterdam UMC Research Institute, Amsterdam, The Netherlands; 12 Mahidol–Oxford Tropical Medicine Research Unit (MORU), Mahidol University, Bangkok, Thailand; 13 Nuffield Department of Medicine, University of Oxford, Oxford, UK; 14 Department of Research and Development, Hamilton Medical AG, Bonaduz, Switzerland

**Keywords:** Randomized controlled trial, Intensive care, ICU, Mechanical ventilation, Invasive ventilation, Automation, Closed-loop, INTELLiVENT–ASV, I–ASV

## Abstract

**Background:**

INTELLiVENT–Adaptive Support Ventilation (ASV) is a fully automated closed-loop mode of ventilation for use in critically ill patients. Evidence for benefit of INTELLiVENT–ASV in comparison to ventilation that is not fully automated with regard to duration of ventilation and quality of breathing is largely lacking. We test the hypothesis that INTELLiVENT–ASV shortens time spent on a ventilator and improves the quality of breathing.

**Methods:**

The “Effects of Automated Closed–loop VenTilation versus Conventional Ventilation on Duration and Quality of Ventilation” (ACTiVE) study is an international, multicenter, two-group randomized clinical superiority trial. In total, 1200 intensive care unit (ICU) patients with an anticipated duration of ventilation of > 24 h will be randomly assigned to one of the two ventilation strategies. Investigators screen patients aged 18 years or older at start of invasive ventilation in the ICU. Patients either receive automated ventilation by means of INTELLiVENT–ASV, or ventilation that is not automated by means of a conventional ventilation mode. The primary endpoint is the number of days free from ventilation and alive at day 28; secondary endpoints are quality of breathing using granular breath-by-breath analysis of ventilation parameters and variables in a time frame of 24 h early after the start of invasive ventilation, duration of ventilation in survivors, ICU and hospital length of stay (LOS), and mortality rates in the ICU and hospital, and at 28 and 90 days.

**Discussion:**

ACTiVE is one of the first randomized clinical trials that is adequately powered to compare the effects of automated closed-loop ventilation versus conventional ventilation on duration of ventilation and quality of breathing in invasively ventilated critically ill patients. The results of ACTiVE will support intensivist in their choices regarding the use of automated ventilation.

**Trial registration:**

ACTiVE is registered in clinicaltrials.gov (study identifier: NCT04593810) on 20 October 2020.

**Supplementary Information:**

The online version contains supplementary material available at 10.1186/s13063-022-06286-w.

## Background

Invasive ventilation can be a lifesaving intervention, but also has the potential to harm the lung tissue [1, 2] and respiratory muscles [3, 4]. Lung injury may be prevented by using an appropriate, usually low tidal volume (*V*_T_) [5, 6] and sufficient positive end-expiratory pressure (PEEP) [7, 8] resulting in a low driving pressure (ΔP) [9, 10] and a low intensity of ventilation [10, 11]. Respiratory muscle injury may be prevented by early use of supported modes of ventilation and the use of spontaneous breathing trials (SBTs) for timely recognition of extubation readiness [3, 4]. Due to rapidly changing conditions in individual critically ill patients, correct interpretation and timely adjustment of ventilator settings is an extremely challenging and time-consuming task for healthcare professionals. Consequently, ventilator settings are often suboptimal [12–16]. Eventually, this could result in longer ventilation times, which translates in a longer stay in the intensive care unit (ICU).

Ventilator manufacturers have developed diverse types of automated, closed-loop modes of ventilation. Such ventilation modes continuously monitor patients’ status and adapt to their needs, using algorithms to automatically adjust ventilator settings and switch to supported breathing when possible. Currently, INTELLiVENT–Adaptive Support Ventilation (ASV) is one of the most sophisticated forms of automated closed-loop ventilation and acts in both active and passive patients—covering ventilation from intubation and start of ventilation to extubation. This ventilation mode is available in ICU ventilators produced by Hamilton (Hamilton Medical AG, Bonaduz, Switzerland), which are broadly used worldwide.

Small-sized clinical studies have shown INTELLiVENT–ASV to be efficient and safe in diverse groups of critically ill patients [17–23]. However, thus far the studies have been underpowered to demonstrate superiority of INTELLiVENT–ASV over non-automated ventilation with respect to patient-centered outcomes. The aim of the “Effects of Automated Closed–loop Ventilation versus Conventional Ventilation on Duration and Quality of Ventilation” (ACTiVE) study is to compare INTELLiVENT–ASV with conventional ventilation that is not fully automated in critically ill patients with respect to ventilation duration and quality of breathing. The primary hypothesis is that INTELLiVENT–ASV is superior to non-automated conventional ventilation with respect to duration of ventilation. The secondary hypothesis is that INTELLiVENT–ASV improves the quality of breathing in a time frame of 24 h early after start of invasive ventilation.

## Methods

### Objectives and design

**Fig. 1:**
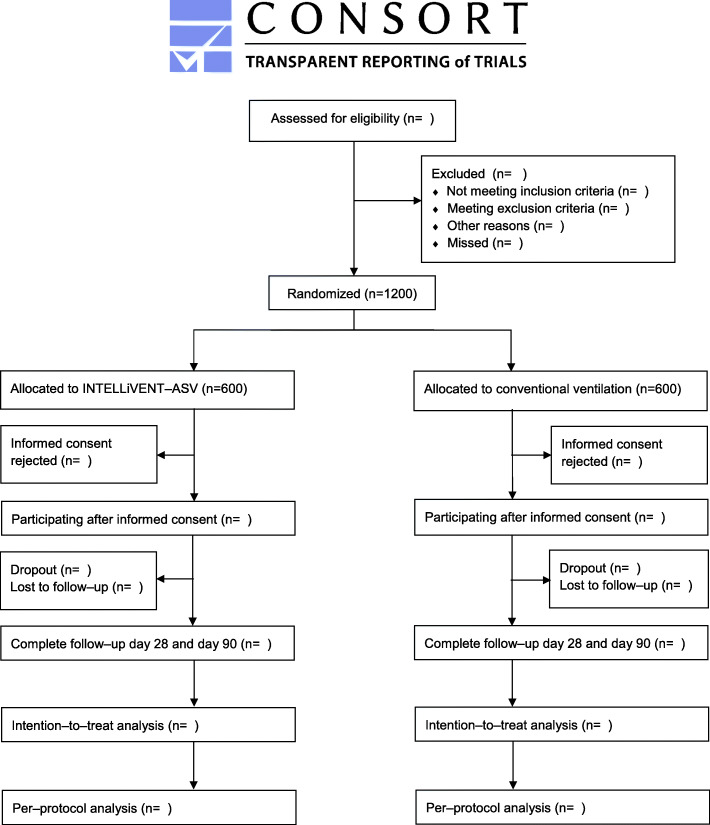
CONSORT flow diagram

The ACTiVE study is an international, multicenter, prospective, two-group, randomized clinical superiority trial in critically ill, invasively ventilated adult ICU patients with an anticipated duration of invasive ventilation of > 24 h. In total, 1200 patients will be recruited in 10 hospitals (*see* Appendix) in Europe and randomly assigned to one of the two to be tested ventilation strategies (*see* Consolidated Standards of Reporting Trials flow diagram (CONSORT) [24] in Fig. 1).

ACTiVE tests the premise that, in patients who need invasive ventilation because of a critical condition, INTELLiVENT–ASV increases the number of days free from invasive ventilation and alive at day 28 (VFDs). One secondary aim is to test whether INTELLiVENT–ASV improves quality of breathing, expressed as the proportion of breaths within lung-protective margins [22], in a time frame of 24 h early after start of invasive ventilation.

ACTiVE has been approved by the Institutional Review Board (IRB) of the Amsterdam UMC, location “AMC,” Amsterdam, the Netherlands. The ACTiVE study is registered at clinicaltrials.gov on October 20, 2020 (study identifier NCT04593810).

### Study population

Local investigators of participating ICUs screen invasively ventilated patients with an anticipated duration of invasive ventilation of > 24 h.

Patients participating in another interventional trial using similar endpoints and patients previously randomized in this study are not eligible. Patients who have received invasive ventilation in the ICU for > 1 h after ICU admission or start of ventilation and patients who received invasive ventilation for > 6 h directly preceding the current ICU admission are excluded. Patients aged < 18 years, with confirmed or suspected pregnancy, who are morbidly obese (body mass index > 40 kg/m^2^), and receiving or planned to receive veno-venous, veno-arterial or arterio-venous extracorporeal membrane oxygenation (ECMO) are excluded from participation. Other exclusion criteria are unavailability of INTELLiVENT–ASV (i.e., no ventilator available with this ventilation mode), recent pneumectomy or lobectomy, premorbid restrictive pulmonary disease, unreliable pulse oximetry (i.e., secondary to carbon monoxide poisoning or sickle cell disease), any neuromuscular diagnosis that can prolong duration of mechanical ventilation (e.g., Guillain–Barré syndrome, amyotrophic lateral sclerosis, multiple sclerosis, myasthenia gravis, or high spinal cord lesion), and no written informed deferred consent from the patient or substitute decision-makers.

### Standard ventilation management

Patients in both groups are ventilated according to local guidelines. Doctors and nurses are responsible for setting and adjusting the ventilator, according to standard clinical practice, and for checking patients’ extubation readiness.

Patients are extubated if standard extubation criteria are fulfilled: patient awake and responsive/cooperative, adequate cough reflex, normal body temperature (rectal temperature > 36.0 °C and < 38.0 °C), adequate oxygenation (ratio of partial pressure of arterial oxygen and fraction of inspired oxygen (PaO_2_/FiO_2_) > 150 mmHg with FiO_2_ ≤ 40%), hemodynamically stable (systolic blood pressure 90 to 160 mmHg and heart rate 40 to 140/bpm with no uncontrolled arrhythmia and no vasopressor/low dosage vasopressor), and adequate lung function (respiratory rate < 35 breaths/minute).

### Intervention

In patients assigned to the “INTELLiVENT–ASV” group, the ventilator is switched to this fully automated mode as soon possible, usually < 1 h after the start of ventilation in the ICU. Patient or lung condition is chosen if applicable (i.e., “acute respiratory distress syndrome (ARDS),” “Chronic Hypercapnia,” and “Brain injury”). If needed, targets zones for end-tidal carbon dioxide (etCO_2_) and saturation of arterial oxygen (SpO_2_) are adjusted. It is advised to enable Quick Wean, a function designated to automate and standardize the weaning process, in all patients. The use of the automated Spontaneous Breathing Trial (SBT) function is left to the discretion of the clinician.

Patients assigned to the “conventional ventilation” group are ventilated with a non-automated mode, e.g., volume-controlled (VCV) or pressure-controlled ventilation (PCV), and pressure support ventilation (PSV), depending on patient’s activity. None of the following automated modes of ventilation is allowed at any time, Neurally Adjusted Ventilatory Assist (NAVA) (Maquet, Getinge Group, Rastatt, Germany), SmartCare/PS (Dräger, Lubeck, Germany), Proportional Assist Ventilation (PAV) (Maquet, Getinge Group, Rastatt, Germany), or the predecessor of INTELLiVENT–ASV named ASV (Hamilton Medical, Bonaduz, Switzerland). In all patients who receive controlled ventilation (i.e., VCV or PCV), three times a day, it should be checked whether the patient can accept supported ventilation (i.e., PSV); this should also be tried when the patient shows respiratory muscle activity during assist ventilation, or in case of patient–ventilator asynchrony. Patients can be subjected to SBTs using either a T-piece or ventilation with minimal support (pressure support level < 10 cm H_2_O). Detailed information on ventilator settings and decisions are provided in Table 1.

**Table 1:** Ventilator settings

	INTELLiVENT–ASV	Conventional ventilation
Settings	Enter patient’s gender and length (measured, not estimated) into the ventilator. Activate SpO_2_ and etCO_2_ sensors and select the INTELLiVENT mode. Set %MinVol, PEEP/CPAP, and oxygen controllers on “Automatic.” If applicable, patient condition is chosen (i.e., “ARDS,” “Chronic Hypercapnia,” or “Brain injury”). Select targets zones for etCO_2_ and SpO_2_ and select default alarm limits. Adjust targets for etCO_2_ and SpO_2_ when the results of the first arterial blood gas analysis are available	Enter patient’s gender and length (measured, not estimated) into the ventilator. Use any non-automated ventilation mode, e.g., (S)CMV, P-CMV and SPONT, depending on patient’s activity. Do not use semi or fully automated ventilation modes at any time (including the predecessor of INTELLiVENT–ASV named ASV)
Weaning	Enable the “Quick Wean” function	Check three times a day whether the patient accepts supported ventilation. Attempt supported ventilation when respiratory muscle activity is seen during assist ventilation, or in case of patient–ventilator asynchrony
SBT	Automated SBT function can be used (left to discretion of the clinician)	SBTs using a T-piece or ventilation with minimal support (pressure support < 10 cm H2O). SBT is successful when respiratory rate < 35/breaths/min, SpO_2_ > 90%, increase < 20% of HR and BP without anxiety or diaphoresis (for at least 30 min)

Abbreviations: *SpO*_*2*_ saturation of peripheral oxygen, *etCO*_*2*_ end-tidal carbon dioxide, *%MinVol* percentage of minute ventilation, *PEEP* positive end-expiratory pressure, *CPAP* continuous positive airway pressure, *ARDS* acute respiratory distress syndrome, *(S)CMV* synchronized controlled mandatory ventilation, *P-CMV* pressure-controlled mandatory ventilation, *SPONT* spontaneous breathing, *ASV* adaptive support ventilation, *SBT* spontaneous breathing trial, *HR* heart rate, *BP* blood pressure

If there is any concern about patient’s safety, the ventilation settings can be changed at any time. A ventilation mode not according to the randomization arm for a period longer than 50% of the duration of ventilation is considered a protocol deviation, and the reason for the deviation must be reported. Protocol deviations are verified by the study monitor and reported and discussed with the Data Safety Monitoring Board (DSMB).

### Standard procedures beyond ventilator management

Tracheostomy is only to be performed on strict indications and preferably not earlier than 10 days after intubation. It can be considered in case of expected duration of ventilation > 14 days, prolonged or unsuccessful weaning, airway protection, severe ICU-acquired weakness based on clinical judgment, repeatedly failed extubations, or pre-existent diminished pulmonary reserves. Weaning with a tracheostomy follows local guidelines.

Sedation strategies follow local guidelines. The use of analgo-sedation over hypno-sedation is favored, as well as bolus over continuous infusion of sedating agents, and the use of sedation scores. The level of sedation should be determined at least three times per day. The goal of sedation is to achieve patient’s comfort and, by minimizing agitation, stress, and fear, to reduce oxygen consumption and physical resistance to daily care.

If consistent with national protocol, selective decontamination of the digestive tract (SDD) should be applied in all patients who are expected to need ventilation for longer than 48 h and/or are expected to stay in ICU for longer than 72 h.

Thrombosis prophylaxis is indicated for all patients who are not treated with anticoagulants and are given according to local guidelines.

Fluid balance should target normovolemia and a diuresis of ≥ 0.5 ml/kg/h. Crystalloid infusions are generally preferred. As soon as possible after ICU admission, a hypo-caloric, protein-rich diet (1.2–1.7 g/kg bodyweight/24 h) should be started, preferably via enteral nutrition. Additional parenteral nutrition can be started if optimal protein intake cannot be reached within 4 days. If stomach retention occurs, administration of prokinetic drugs followed by a duodenal tube can be used, according to local guidelines.

### Minimization of bias

Randomization is performed by the local investigators using a dedicated and password-protected randomization tool in Castor Electronic Data Capture (EDC). Castor EDC generates the allocation sequence. Stratification per center and permuted blocks of different block sizes are used, with a maximum block size of 8. Local investigators enroll participants and assign them in a 1:1 ratio to the “INTELLiVENT–ASV” or the “conventional ventilation” arm. Due to the nature of the intervention, blinding of personnel is not possible; all analyses will be performed in a blinded fashion.

### Study endpoints

The primary endpoint of ACTiVE is the number of days free from invasive ventilation and alive at day 28 (VFD–28), defined as the number of days from day 1 to day 28 after randomization that the patient is alive and breathes without assistance of the mechanical ventilator. A patient must be free from invasive ventilation for at least 24 h to have one VFD–28. In case of multiple extubations within day 28, only the last extubation will be considered for this endpoint. Patients who die before day 28 or are invasively ventilated for longer than 28 days are assigned to have no VFD–28.

One key secondary endpoint is quality of breathing, defined as the time spent within predefined zones of ventilation in a time frame of 24 h early after start of invasive ventilation. Different zones will be specified for certain patient categories (e.g., patients classified by the attending doctors as having acute respiratory distress syndrome (ARDS) versus chronic obstructive pulmonary disease, and patients with a low compliance and oxygenation disturbances [suggestive of ARDS] versus patients with a high compliance and hypercapnia [suggestive of COPD]). This analysis is restricted to patients in centers where these data can be collected from an available communication port at the ventilator.

Other endpoints are duration of ventilation in survivors, ICU and hospital LOS, and mortality in the ICU and hospital, and at day 28 and day 90. We will also report incidences of development of ARDS, severe hypoxemia and hypercapnia, severe atelectasis and pneumothorax, ventilator-associated pneumonia (VAP), use of rescue therapies for severe hypoxemia or severe atelectasis (recruitment maneuver, prone positioning, or bronchoscopy for opening atelectasis), and extubation failure (reintubation within 24 h).

We will report maximal inspiratory pressure (MIP) within 72 h after extubation at centers that can collect these data and quality of life at day 28.

### Study visits and data collection

Demographic and baseline data, as well as data on disease severity, are collected at ICU admission and within the first 24 h thereafter. The data collected are the following: age, gender, height, weight, reason for ICU admission and for ventilation support, cause of respiratory failure, the Acute Physiology and Chronic Health Evaluation (APACHE) II or IV score, or the Simplified Acute Physiology Score (SAPS) II.

Data on clinical outcome variables (described below and in the Appendix) are collected daily until day 28, ICU discharge, or death, whatever comes first. Data on ICU and hospital length of stay (LOS), location of the patient (ICU, hospital, other facility, or home), and life status (alive or deceased) are assessed on day 28 and day 90. The following variables are collected daily: respiratory status, ‘duration of ventilation according to randomization (hours), and rationale for adjustment, if mode according to randomization is changed’ as follows ‘(...) intubation status (if extubated: time of extubation, MIP in a subset of patients, and if self-extubation or extubation failure occurred), duration of ventilation according to randomization (hours), and rationale for adjustment, if mode according to randomization is changed, tracheostomy status (...)’ tracheostomy status (in case of tracheostomy: time of tracheostomy and weaning status), development of pulmonary complications (ARDS, severe hypoxemia or hypercapnia, VAP, severe atelectasis, pneumothorax), need for rescue therapies for severe hypoxemia or severe atelectasis (recruitment maneuver, prone positioning, bronchoscopy for opening atelectasis), and development of non-pulmonary complications (ICU-acquired weakness and delirium).

The following ventilation parameters are collected within the hour before and at 1 h after randomization, and every day at a fixed time point until day 5 or liberation from the ventilatory support, whatever comes first: mode of ventilation, tidal volume, respiratory rate, level of PEEP, FiO_2_, SpO_2_, etCO_2_, peak pressure or maximum airway pressure, plateau pressure, and level of pressure support above PEEP. ICU-related therapy variables to collect daily until day 5 or liberation from the ventilatory support include arterial blood gas analysis (once daily), daily fluid balance, daily dose of sedatives, Sequential Organ Failure Assessment (SOFA) score.

**Fig. 2:**
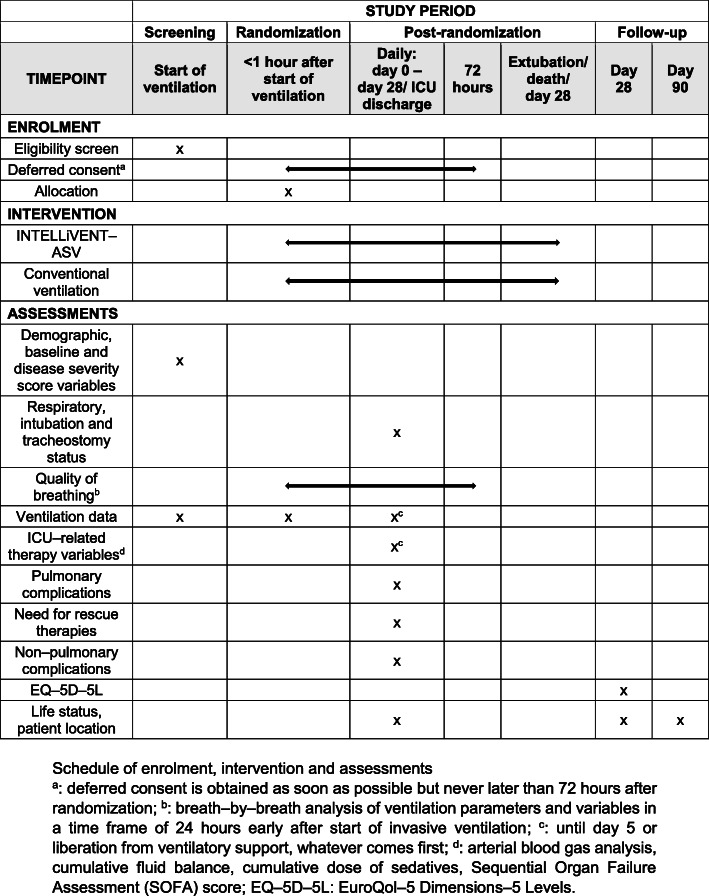
Schedule of enrollment, intervention, and assessments

The quality of life questionnaire EuroQol–5 Dimensions–5 Levels (EQ–5D–5 L) [25] is administered at day 28: the questionnaire is sent by post or administered by telephone (Appendix). Day 90 is defined as the last day of follow-up. The schedule of enrollment, intervention, and assessments are summarized in Fig. 2.

### Deferred consent

For this study, we include patients using a deferred informed consent since we explicitly want to randomize and start ventilation accordingly within 1 h after intubation, or within 1 h after ICU admission if ventilation was initiated in the emergency or in the operation room. Nevertheless, written informed consent from the legal representative must be obtained as soon as possible, but always within 72 h after randomization. The substitute decision-maker is informed verbally by local researchers and by a patient information letter. If informed consent is not obtained within this time window, or if the substitute decision-maker denies participation within this time frame, the patient is excluded and data is no longer used. If consent is obtained from the substitute decision-makers, as soon as the patient is recovered, informed written consent is asked from the patient.

If the substitute decision-maker is not able to visit the hospital for whatever reason, the investigator can contact the representative by telephone to obtain substitute consent within a maximum timeframe of 72 h; after verbal consent an email is sent to the substitute decision-maker asking to confirm the continuation of study participation. At a later time, a complete informed consent is sent to the substitute decision-maker, which must be signed and returned.

### Study dropouts and missing data

Participation in the ACTiVE study is voluntary. The number of dropouts is expected to be very low. The patient or patient’s relatives can withdraw consent for collecting study data and for participation in the study at any time during the trial and without giving a reason for this. Dropout patients will not be replaced. No or minimal losses to follow-up for the primary and secondary outcomes are anticipated. Lost to follow-up cases due to withdrawal of consent or for other reasons will be excluded form analysis. If more than 1% of missing data will be found for the primary outcome, a sensitivity analysis using multiple imputations and estimating-equation methods will be carried out.

### Handling of data

Patient identifying personal data is replaced by an assigned patient identification code. The codebook is stored digitally, and in paper, the paper version behind a lock and the digital form encrypted with a double password. All data will be stored for the length of the study and for 15 years afterwards.

The results of ACTiVE will be published in scientific journals and used for national and international guideline. A summary of the results will be placed on clinicaltrials.gov to inform participants.

### Sample size calculation

We will include a total of 1200 patients. The sample size is based on the hypothesis that INTELLiVENT–ASV will shorten ventilation duration by 1.5 days with no changes in mortality rate. Based on previously performed studies in which the mean number of VFD–28 was 20 ± 9 days [26, 27], a sample of 1200 patients (600 in each treatment group) is needed to have beta of 80% power and a two-tailed alpha of 0.05, to detect a mean between-group difference of 1.5 VFD–28, allowing a dropout rate of 5%.

### Statistical analysis

A detailed statistical analysis plan will be updated, finalized, and made available before the inclusion of the last patient. The statistical analysis will be based on the intention-to-treat principle, with patients analyzed according to their assigned treatment arms, except for cases lost to follow-up, or patients who are withdrawn due to lack of deferred informed consent. In addition, we will conduct per-protocol analyses, which only considers those patients who completed the treatment according to the originally allocated protocol.

When appropriate, statistical uncertainty will be expressed by the 95% confidence levels. *P*-values of 0.05 will be used for statistical significance. For the experimental and control arms, continuous normally distributed variables will be expressed by their mean and standard deviation or, when not normally distributed, as medians and their interquartile ranges. Categorical variables will be expressed as frequencies and percentages. The number of VFD–28 data will be presented as a median difference and a two-sided 95% confidence interval. Analyses of the primary and secondary outcomes will be described in detail in the statistical analysis plan.

### Trial organization

The steering committee consists of three principal investigators, three trial coordinators, two international experts in invasive ventilation who contributed to the design of the study protocol, and the local investigators at participating study sites. The steering committee remains responsible for the interpretation of the data and drafts the final report that will be approved by all investigators.

An independent DSMB, consisting of four individuals with extensive clinical research experience in the field of mechanical ventilation (Prof. Bronagh Blackwood, PhD; Prof. Carol Hodgson, PhD; Prof. Ignacio Martin–Loeches MD PhD; Prof. Frank van Haren MD PhD), overviews the study conduct and the possible side effects of the study treatment. The first meeting must be soon after the start of the study; subsequent to this meeting, the DSMB meets every 6 months. Ad hoc meeting of the DSMB may be called at any time by one of the principal investigators or the DSMB chair. This study compares two ventilation strategies that are currently widely used in standard care. For this reason, serious adverse events (SAEs) related to the study are not to be expected. Two statisticians, one blinded and one unblinded, report every 6 months to the DSMB the secondary endpoints of this trial, which incorporate ventilation-specific complications, to monitor safety of both treatment strategies. These endpoints are specified per study arm in a line listing without disclosing the specific arms. The same line listing is presented to the IRB. The ventilation-specific complications include ICU mortality, incidence of self-extubation, incidence of ARDS, incidence of severe atelectasis, incidence of pneumothorax, incidence of VAP, and incidence of severe hypoxemia and of severe hypercapnia (see the Appendix for definitions).

This study is an investigator-initiated trial, sponsored by the Amsterdam UMC, location “AMC.” The study is funded by “The Netherlands Organization for health Research and Development” (ZonMw). The sponsor, in accordance to section 10, subsection 4, of the Medical Research Involving Human Subjects Act (WMO), can halt the study at any time if there is sufficient ground that continuation of the study will jeopardize subject’s health or safety. Hamilton Medical AG, the manufacturer of the ventilators that can be used for INTELLiVENT–ASV, had no role in design and will not have a role in reporting of the study.

An independent monitor performs clinical trial monitoring according to the approved monitor plan. On-site monitoring comprises controlling the presence and completeness of the trial master file (TMF) and investigator site file (ISF) and the informed consent forms, and source data check is performed as described in the monitoring plan. Remote monitoring is performed between the routine on-site monitoring visits to signal early aberrant patterns, issues with consistency, credibility, and other anomalies. Centralized initiation meetings are organized before sites can start including patients.

## Discussion

ACTiVE tests the hypothesis that INTELLiVENT–ASV is superior to conventional ventilation which is not fully automated with respect to duration of ventilation and the quality of breathing. Thus far, there have been no randomized clinical trials that were sufficiently powered to answer this question. We are aware of one multicenter randomized clinical trial, named EASiVENT, that will test a comparable hypothesis—a study that enrolls patients in the USA, Switzerland and, France (Clinical trials.gov NCT04400643). EASiVENT, however, is smaller than the ACTiVE study, as depending on the results of an interim analysis this study will include 288 to a maximum of 576 patients.

The secondary hypothesis tested is that this fully automated ventilation mode improves the quality of breathing. For this, we use an approach that was used before in a study that compared INTELLiVENT–ASV with conventional ventilation in the postoperative phase in cardiac surgery patients [22]. In line with that study, we will classify each breath as “optimal,” “acceptable,” or “critical” according to the tidal volume, the maximum airway pressure, the end-tidal carbon dioxide level, and the saturation of arterial oxygen [17]. These data will only be available for patients in centers where granular ventilation can be collected through so-called memory boxes connected to the ventilator. It is expected that we will have these data for at least 100 patients—this number is sufficient to test the secondary hypothesis.

In critically ill patients, it is usually recommended to use an appropriate low *V*_T_, to titrate PEEP and inspired fraction of oxygen by means of a lower or higher PEEP/FiO_2_ table, to target a lower driving pressure, and to avoid both hyperoxia and hyperoxemia. This can be a challenge, especially when ICU doctors and nurses are less experienced in invasive ventilation. Besides, ICU doctors and nurses often do not have sufficient time to adjust ventilator settings, and at best do so every hour. Automation of ventilation uses breath-by-breath adjustments for safer and more efficient ventilation [22]. With that, the risk for ventilator-induced lung injury could be reduced, eventually leading to better patient outcomes. In addition, automated modes have the potential to reduce the amount of ventilator alarms doctors and nurses have to respond to, further reducing time needed to set, or in this case adjust the ventilator settings. With the expected growing numbers of critically ill patients that need invasive ventilation, and the faired shortages of nurses available at the bedside, automation of ventilation becomes increasingly important.

We use a clinically relevant patient-centered outcome, the number of days alive and free from invasive ventilation at day 28. This composite endpoint is chosen because it reflects both duration of ventilation in surviving patients, and also mortality, which remains high in the ICU. Of note, we do not expect a significant difference in mortality between the groups. Nevertheless, we consider this composite outcome a better indicator of the potential effect on actual duration of ventilation, which could otherwise be difficult to distinguish given the high mortality rates in the two groups.

Ventilation in both groups is highly standardized, especially with respect to weaning as this has an effect on the primary endpoint of the study. Standard care follows strict local clinical guidelines. ACTiVE aims at minimizing bias by using concealed allocation and an intention-to-treat analysis with a pragmatic protocol that can be strictly adhered to. ACTiVE is performed in both community as teaching hospitals in different countries of Europe, making the results generalizable.

In summary, ACTiVE will be among the first sufficiently powered multicenter, randomized clinical trials that test whether INTELLiVENT–ASV is superior to conventional ventilation with respect to duration of ventilation and quality of breathing. The results of ACTiVE can support intensivists in their choices regarding the use of automated ventilation in their ICU.

## Trial status

The current approved version of the protocol is version 4, issue date July 2021. ACTiVE is currently recruiting patients. Recruitment started in October 2020 and will be completed in approximately 3 years.

## Supplementary Information

**Additional file 1: Appendix.** List of participating hospitals. Definitions of clinical outcome variables. EQ-5D-5L Quality of Life Questionnaire

**Additional file 2.** A complete checklist of recommended items to address in a clinical trial protocol and related documents according to the ‘Standard Protocol Items: Recommendations for Interventional Trials (SPIRIT) 2013’ [28].

## Data Availability

The datasets of the ACTiVE study and the statistical code will be available upon request to the steering group. The full protocol will be available from the corresponding author on a reasonable request*.*

## Abbreviations

APACHE: Acute Physiology and Chronic Health Evaluation
ARDS: Acute respiratory distress syndrome
ASV: Adaptive support ventilation
CONSORT: Consolidated Standards of Reporting Trials
ΔP: Driving pressure
DSMB: Data Safety Monitoring Board
ECMO: Extracorporeal membrane oxygenation
EDC: Electronic data capture
EQ–5D–5 L: EuroQol–5 Dimensions–5 levels
etCO_2_: End-tidal carbon dioxide
FiO_2_: Fraction of inspired oxygen
GAMLSS: Generalized additive model for location scale and shape
ICU: Intensive care unit
IRB: Institutional Review Board
ISF: Investigator site file
LOS: Length of stay
MIP: Maximal inspiratory pressure
NAVA: Neurally adjusted ventilatory assist
PaO_2_: Partial pressure of arterial oxygen
PAV: Proportional assist ventilation
PCV: Pressure-controlled ventilation
PEEP: Positive end-expiratory pressure
PSV: Pressure support ventilation
SAEs: Serious adverse events
SAPS: Simplified acute physiology score
SBT: Spontaneous breathing trial
SOFA: Sequential organ failure assessment
SPIRIT: Standard Protocol Items: Recommendations for Interventional Trials
SpO_2_: Saturation of arterial oxygen
TMF: Trial master file
VAP: Ventilator-associated pneumonia
VCV: Volume-controlled ventilation
VFD–28: Ventilator-free days and alive at day 28
*V*_T_: Tidal volume
WMO: Medical Research Involving Human Subjects Act
ZonMw: The Netherlands Organization for health Research and Development
