# Supplementary material for: Effect of Automated Closed-loop ventilation versus convenTional VEntilation on duration and quality of ventilation in critically ill patients (ACTiVE) – study protocol of a randomized clinical trial
Source: Trials. 2022 Apr 23;23:348. doi: 10.1186/s13063-022-06286-w (PMC9034629; doi:10.1186/s13063-022-06286-w)
Supplement: Supplementary file 1 — Additional file 1: Appendix. List of participating hospitals. Definitions of clinical outcome variables. EQ-5D-5L Quality of Life Questionnaire [file 13063_2022_6286_MOESM1_ESM.pdf]

APPENDIX

LIST OF PARTICIPATING HOSPITALS

- Amsterdam University Medical Centers, location Academic Medical Center, Amsterdam, The Netherlands
- Canisius Wilhelmina Hospital, Nijmegen, The Netherlands
- Catharina Hospital Eindhoven, Eindhoven, The Netherlands
- Diakonessenhuis, Utrecht, The Netherlands
- Elisabeth–TweeSteden Hospital, Tilburg, The Netherlands
- Flevo Hospital, Almere, The Netherlands
- Leiden University Medical Centre, Leiden, The Netherlands
- Reinier de Graaf Hospital, Delft, The Netherlands
- San Martino Polyclinic Hospital, IRCCS for Oncology and Neurosciences, Genova, Italy
- San Matteo Polyclinic Foundation, University of Pavia, Pavia, Italy

DEFINITIONS OF CLINICAL OUTCOME VARIABLES

- Ventilator–free days and alive at day 28 (VFD–28):
  - VFD–28 = 0 if subject dies within 28 days of mechanical ventilation
  - VFD–28 = 28 – x if successfully liberated from ventilation x days after initiation
  - VFD–28 = 0 if the subject is mechanically ventilated for ≥ 28 days
- Moderate or severe ARDS: all following criteria must be met
  - Bilateral opacities on chest radiograph or chest CT
  - PaO_2/FiO_2 ratio <200 mm Hg or <100 mm Hg with PEEP ≥ 5 cm H_2O
  - Respiratory failure not fully explained by cardiac failure or fluid overload
  - Onset 48 hours after randomization
- Severe hypoxemia: PaO_2 < 7.3 kPa
- Severe hypercapnia: $PaCO_2$ > 7.33 kPa combined with a pH < 7.35
- Ventilator Associated Pneumonia (VAP): new or progressive radiographic infiltrate 48 hours after randomization with a positive sputum culture plus at least one of the following:
  - fever (tympanic temperature > 38.5 °C)
  - leukocytosis or leukopenia (leukocytes < 4 or > 10.5 $\cdot 10^9 \cdot L^{-1}$)
- Severe atelectasis: at least complete lobar atelectasis of a lung determined on chest radiograph or chest CT by a radiologist
- Pneumothorax: air in the pleural space created after randomization determined on chest radiograph or chest CT by a radiologist
- Recruitment manoeuvre: increase of inspiratory pressure or the level of PEEP for at least 40 seconds

- Bronchoscopy for opening atelectasis: bronchoscopy performed with indication to open atelectasis or when the pulmonologist noticed that he/she has removed sputum plugs during bronchoscopy
- ICU–acquired weakness: mean MRC (Medical Research Council) score < 4 if not all 12 muscle groups were scored or a sum score < 48 out of 60 if all 12 muscle groups were scored. MRC score:
    - 0 = no muscle contraction
    - 1 = some muscle contraction, no joint movement
    - 2 = joint movement with gravity
    - 3 = joint movement against gravity
    - 4 = joint movement against some resistance
    - 5 = normal force

EQ-5D-5L Quality of Life Questionnaire

MOBILITY
I have no problems in walking about □
I have slight problems in walking about □
I have moderate problems in walking about □
I have severe problems in walking about □
I am unable to walk about □

SELF-CARE
I have no problems washing or dressing myself □
I have slight problems washing or dressing myself □
I have moderate problems washing or dressing myself □
I have severe problems washing or dressing myself □
I am unable to wash or dress myself □

USUAL ACTIVITIES (e.g. work, study, housework, family or leisure activities)
I have no problems doing my usual activities □
I have slight problems doing my usual activities □
I have moderate problems doing my usual activities □
I have severe problems doing my usual activities □
I am unable to do my usual activities □

PAIN / DISCOMFORT
I have no pain or discomfort □
I have slight pain or discomfort □
I have moderate pain or discomfort □
I have severe pain or discomfort □
I have extreme pain or discomfort □

ANXIETY / DEPRESSION
I am not anxious or depressed □
I am slightly anxious or depressed □
I am moderately anxious or depressed □
I am severely anxious or depressed □
I am extremely anxious or depressed □

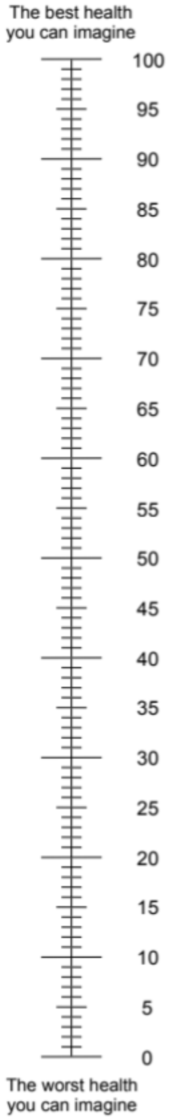

We would like to know how good or bad your health is TODAY.
This scale is numbered from 0 to 100.
100 means the best health you can imagine. 0 means the worst health you can imagine.
Mark an X on the scale to indicate how your health is TODAY.
Now, please write the number you marked on the scale in the box below.

YOUR HEALTH TODAY = ☐
